# Transmission of Influenza A in a Student Office Based on Realistic Person-to-Person Contact and Surface Touch Behaviour

**DOI:** 10.3390/ijerph15081699

**Published:** 2018-08-09

**Authors:** Nan Zhang, Yuguo Li

**Affiliations:** Department of Mechanical Engineering, The University of Hong Kong, Pokfulam Road, Hong Kong, China; zhangnan@hku.hk

**Keywords:** influenza A, airborne, fomite, close contact, infection, surface touch, office, mask, hand-washing

## Abstract

Influenza A viruses result in the deaths of hundreds of thousands of individuals worldwide each year. In this study, influenza A transmission in a graduate student office is simulated via long-range airborne, fomite, and close contact routes based on real data from more than 3500 person-to-person contacts and 127,000 surface touches obtained by video-camera. The long-range airborne, fomite and close contact routes contribute to 54.3%, 4.2% and 44.5% of influenza A infections, respectively. For the fomite route, 59.8%, 38.1% and 2.1% of viruses are transmitted to the hands of students from private surfaces around the infected students, the students themselves and other susceptible students, respectively. The intranasal dose via fomites of the students’ bodies, belongings, computers, desks, chairs and public facilities are 8.0%, 6.8%, 13.2%, 57.8%, 9.3% and 4.9%, respectively. The intranasal dose does not monotonously increase or decrease with the virus transfer rate between hands and surfaces. Mask wearing is much more useful than hand washing for control of influenza A in the tested office setting. Regular cleaning of high-touch surfaces, which can reduce the infection risk by 2.14%, is recommended and is much more efficient than hand-washing.

## 1. Introduction

Influenza is a highly contagious respiratory illness that causes 1,250,000 deaths annually worldwide [1]. It is estimated that influenza A viruses result in the deaths of approximately half a million individuals worldwide every year [2]. Influenza A viruses exist on many surfaces in our daily lives, such as towels in homes and medical cart items in hospitals [3].

Despite human beings’ vast clinical experience, debate about the transmission of influenza continues [4]. The influenza virus is known to be spread from person to person by at least two mechanisms: direct and indirect transfer of respiratory secretions and contact with large droplets that settle onto fomites [5]. Studies have shown that influenza A may be transmitted by inhalation of small airborne particles [6]. Although some evidence was found of animal-to-animal transmission [7], other evidence of person-to-person transmission was based on observational and epidemiological studies [8]. Influenza can also be transmitted by droplet nuclei [9,10]. In addition, other studies have found that the fomite route could be a potential route of influenza transmission [11]. In general, influenza A can be spread via the airborne, droplet and direct and indirect contact routes.

Many simulations of influenza transmission have been conducted in enclosed spaces such as hospitals [12] and air cabins [13], and in large spaces such as cities [14], and even between cities [15]. Large-scale influenza transmission is usually based on macroscopic factors such as human mobility via airplane and other modes of public transport [15]. Microscopic factors such as how people talk or make contact with each other are ignored because of complexity. Most human behaviour, including person-to-person contact and surface touches, are hypothesised even when all transmission routes are considered. However, human behaviour changes with the environment, and large errors may result if all human behaviour is unknown or randomly set. A simulation of influenza A transmission based on realistic data of human behaviour in a confined space is needed to help understand influenza A transmission and to implement effective measures to prevent and control disease.

In this study, we simulated influenza A transmission in a graduate student office by considering three routes: long-range airborne, fomite and close contact (short-range airborne and droplet spray). All student behaviour, including close contact and surface touches in the office, was recorded by video-camera from 9 a.m. to 9 p.m., from 11 to 15 September 2017. The data included more than 3500 close contacts between students and more than 127,000 surface touches. Influenza A transmission was simulated in the office via three routes based on realistic behaviour obtained from these recorded data. We discovered how influenza A virus is transmitted via air, hands, surfaces, mucous membranes and inhalation. We also analysed the efficacy of various strategies for prevention of influenza A via various transmission routes.

## 2. Materials and Methods

We simulated influenza A transmission via three routes (long-range airborne, fomite and close contact) in a graduate student office in China. The office measured 12 × 8.4 × 2.7 m and included 39 students. All surfaces were grouped into six categories (primary surfaces): private (i.e., the student [*Std*]; body parts), students’ belongings (*Bln*), desks (*Dsk*), chairs (*Chr*), computers (*Cpt*) and public facilities (*Pbf*). These primary surfaces were in turn divided into 31 secondary surfaces consisting of 57 types of sub-surfaces with multiple surfaces for each type, giving a total of 1490 basic surfaces (Supplementary Materials: Table S1).

The data for each student’s surface touch behaviour were taken from four video-cameras installed on the office ceiling to monitor student behaviour of close contact and surface touch from 9 a.m. to 9 p.m. on five successive weekdays from 11 to 15 September 2017. All students in the office gave informed consent for inclusion before they participated in the study. Five video image analysis assistants were appointed to process the data second by second, recording all visible touch actions made by every student in the office. A surface touch was defined as any contact between a hand or finger and a solid surface or object lasting for 1 s or longer. The information recorded comprised the onset time, duration, and location of each touch, and details of which student had done the touching, which hand had been used, and which surface had been touched. For quality control during the video image analysis, one author (NZ) verified touch actions at two time points, namely the 15th minute and the 45th minute of each of the 60 h. Verification revealed that 67 out of the 1712 touch actions (3.1%) had been incorrectly recorded by the original video analysers.

In the 5-day study period, 127,052 touches by left and right hands on 1490 coded surfaces were recorded. The surfaces had a total exposure to touch of 1,517,958 person·seconds. The students spent 90.74% of their time in the office touching surfaces [16].

**Figure 1:**
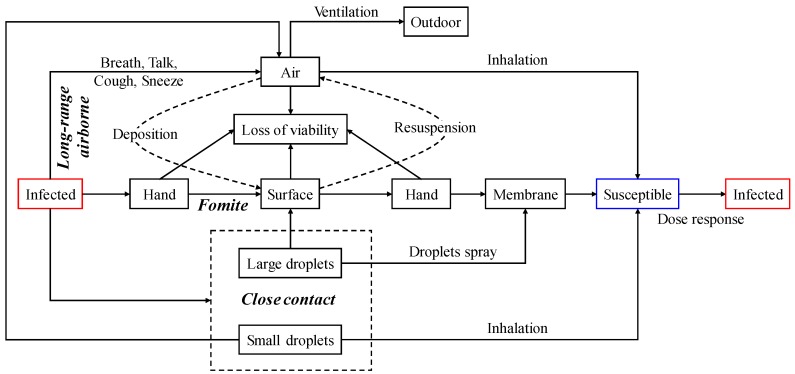
Three routes for influenza A transmission.

In this paper, we used influenza A as an example. Figure 1 illustrates how influenza A is transmitted via the long-range airborne, fomite and close contact routes. When an infected student breathes, talks, coughs or sneezes, the virus is carried in particles and released into the environment. Small droplets disperse in the air. Some floating particles flow outdoors through the ventilation system, some are inhaled by students, some are deposited on surfaces and those still in the air lose viability over time. Large droplets are rapidly deposited on surfaces near the infected student or are directly sprayed onto the hands of the infected person while covering their mouth and nose while coughing or sneezing. The virus will then be transferred to a surface if it is touched by a contaminated hand. A clean hand can also be contaminated if it touches a contaminated surface. Some large droplets with virus on hands can be transferred to the mucous membranes if the student touches his or her own lips, nose or eyes. Some large droplets are resuspended from surfaces to the air because of human activities such as walking. In addition, viruses on hands and surfaces also lose viability over time. Moreover, when an infected student speaks face-to-face with other students, particles are sprayed from the mouth of the infected student. Some small particles will be directly inhaled via the respiratory tract by students who speak with the infected student, and some large particles might be rapidly deposited on their mucous membranes. The inhalation dose and intranasal dose of susceptible students gradually increase, and each student’s infection risk can be calculated based on the dose-response parameters of influenza A.

All students go to lunch and dinner between 12 a.m. and 1 p.m. and between 5 p.m. and 6 p.m., respectively, every day. In real conditions, all students do not stay in the office all the time. To reduce the randomness caused by their stay duration, all students are assumed to remain in the office from 9 a.m. to 9 p.m. except for lunch and dinner time.

### 2.1. Parameter Setting

The US Centers for Disease Control and Prevention (CDC) have defined three influenza transmission routes: long-range airborne, fomite (direct and indirect) and close contact (Table 1). Only particles with a diameter of less than 10 μm can penetrate the lungs [17]. Pathogens carried in particles with an aerodynamic diameter ($d_{a}$) between 10 and 100 μm are inspired if a person is facing the patient at close range during an expiratory event such as a cough or a talk [18]. More than 99% of the pathogens emitted in a cough are carried by particles with an aerodynamic diameter $d_{a}$ > 100 μm [19], and these particles tend to settle rapidly on surfaces near the point of emission [18].

At the beginning of the simulation, there are 39 students in the office, i.e., one infected student (*P*_0_) and 38 susceptible students (*P*_1_–*P*_38_). They touch surfaces and talk with each other based on realistic data on surface touch and interpersonal contacts obtained from the video analysis. In each simulation, all students will select an action, such as with surfaces they touch and with whom they talk, from the database (collected surface touch and close contact data) at each time step (second).

### 2.2. Long-Range Airborne

Particles of various sizes ae generated by activities such as breathing, talking, coughing and sneezing. We assumed that particles ($d_{a}$ < 10 μm) uniformly floated in the air of the office. The deposition of these small particles is slight because it requires around an hour in a steady environment [21,22]. In an office setting, the deposition rate will be reduced because of frequent walking by the students. We assumed that the deposition rate was 0.1 min^−1^, which means that 10% of the particles’ volume is deposited uniformly on all surfaces per minute.

Because the difference in the association between the size and number of particles from previous researchers is large, we use the average values from previous studies to assess the total virus quantity in small particles. Summarising the previous studies [23,24,25,26,27,28,29], the total volume of small particles ($d_{a}<10\;\mu m$) by breathing ($V_{vb}^{\prime }$), talking ($V_{vt}^{\prime }$) (counting from 1 to 100, which takes about 100 s), coughing ($V_{vc}^{\prime }$) and sneezing ($V_{vs}^{\prime }$) are estimated as $1.02\times 10^{-10}\;\mathrm{mL}$, $1.47\times 10^{-7}\;\mathrm{mL}$, $1.65\times 10^{-7}\;\mathrm{mL}$ and $1.27\times 10^{-6}\;\mathrm{mL}$, respectively. The total volume of large particles ($d_{a}>10\;\mu m$) generated by breathing ($V_{vb}^{\prime \prime }$), talking ($V_{vt}^{\prime \prime }$), coughing ($V_{vc}^{\prime \prime }$) and sneezing ($V_{vs}^{\prime \prime }$) are estimated as 0 mL, $5.15\times 10^{-3}\;\mathrm{mL}$, $6.15\times 10^{-3}\;\mathrm{mL}$ and $4.75\times 10^{-2}\;\mathrm{mL}$, respectively. Here, we assumed that the size distribution of particles generated by coughing and sneezing was the same. People take an average of 15 breaths per minute, so a breath takes around 4 s [30]. The average frequencies of coughing and sneezing episodes for an influenza A infected person are 22/h [31] and 5/h [32] respectively. A cough takes 1 s, and a sneeze is assumed to take 3 s. Our recorded data show that students in the office spent an average of 10% of their time talking with others. Therefore, the frequency of breathing ($f_{b}$), talking ($f_{t}$), coughing ($f_{c}$) and sneezing ($f_{s}$) of an infected person are 21,600 times, 0.8 h (two students on average share the time during talking), 352 times and 80 times per day (talking, coughing and sneezing during 16 h except 8-h sleep). The total volume of droplets generated by an infected person per day ($V_{V}$) is calculated by Equation (1).

(1) $$V_{v}=V\prime_{v}+V\prime \prime_{v}=\sum_{i=1}^{i=4}\left(V\prime_{vi}+V\prime \prime_{vi}\right)\cdot f_{i}\approx 6.11\;\mathrm{mL}/\mathrm{day}$$

where *i* = 1 to 4 shows the human activities of breathing, talking, coughing and sneezing, respectively; $V\prime_{v}$ and $V\prime \prime_{v}$ are the total volume of small and large droplets generated by an infected person per day, respectively.

We assumed that an infected person’s initial viral shedding rate is around $1.5\times 10^{7}\;\mathrm{TCID}50/\mathrm{day}$ [19,24,25,31,33], and it changes with time and the severity of infection. Because the amount of virus in a particle is roughly proportional to the particle’s volume [34], we considered the virus concentrations in all particles to be the same. The virus concentration ($C_{v}$) in all particles is then obtained ($C_{v}\;=1\times 10^{6.39}\;\mathrm{TCID}50/\mathrm{mL}$). This value is between the virus concentration in nasopharyngeal fluid ($10^{2}–10^{7}\;\mathrm{TCID}50/\mathrm{mL}$) measured by Douglas [35] and that in saliva ($10^{4}–10^{8}\;\mathrm{TCID}50/\mathrm{mL}$) estimated by Nicas [36].

The ventilation rate ($V_{AC}$) in offices is usually set to 1 ACH [37]. The respiratory rate ($R_{R}$) of a person is around 0.38 m^3^/h (0.11 L/s) [38]. The inactivation rate ($\mu_{a}$) of influenza A in aerosols in the air changes with the relative humidity (RH%) and temperature [39]. We assumed that the relative humidity and temperature in the office were around 50% and 20 °C to 24 °C, respectively; they were not measured. The inactivation rate ($\mu_{a}$) of influenza A in aerosols in the air is 13.9 day^−1^ [40].

If a susceptible person inhales aerosol that contains influenza A viruses, he or she has a probability of being infected. The infection probability depends on the total inhalation quantity of the virus.

(2) $$\;IR_{I}=1-\exp\left(-\alpha_{R}\times D\right)$$

where $IR_{I}$ is the infection risk (probability to be infected), D is the respiratory dose, $\alpha_{R}$ is the estimated dose-response parameter for exposure to the respiratory tract distal to the head airways from the respirable particle inhalation study, and $\alpha_{R}=0.18\;\mathrm{TCID}50^{-1}$ is obtained by influenza A2/Bethesda/10/63 virus aerosol in humans [36].

All values mentioned above are listed in Table 2.

### 2.3. Fomite

Large droplets rapidly settle on the ground [41]. In the simulations, we considered that droplets larger than 10 μm ($d_{a}>10\;\mu m$) do not remain airborne long enough to become respirable [19]. When an infected student talks, coughs or sneezes, areas 1-m in front of and 0.5-m on both sides of the patient will be contaminated [29]. We assumed that half of the particles are deposited on surfaces that are touched by the infected persons themselves and that the remaining 50% of particles are randomly distributed on the surfaces near the infected persons when they talk, cough and sneeze. If no surface is touched, half of the particles are deposited on the infected student’s desktop if he or she sits on his or her own chair; otherwise, half of the particles are deposited on the floor. According to the analysis of human touch behaviour in an office, the touch frequency for each surface is listed in Table 3, and the type and area of each surface are also listed.

When a hand touches a surface, the total quantity of virus (TCID50) on the hand and on the surface can be calculated based on Equations (3) and (4).

(3) $$\frac{dV_{h}\left(t\right)}{dt}=R_{sh}\cdot A_{c}\cdot \frac{V_{s}}{A_{S}}-R_{hs}\cdot A_{c}\cdot \frac{V_{h}}{A_{h}},$$

(4) $$\frac{dV_{s}\left(t\right)}{dt}=R_{hs}\cdot A_{c}\cdot \frac{V_{h}}{A_{h}}-R_{sh}\cdot A_{c}\cdot \frac{V_{s}}{A_{S}},$$

where $V_{h}\left(t\right)$ and $V_{s}\left(t\right)$ are the total quantity of virus (TCID_50_) on a hand and a surface at time *t* caused by surface touch, respectively; $R_{sh}$ and $R_{hs}$ are the virus transfer rates from surfaces to hands and from hands to surfaces, respectively; $A_{s}$, $A_{h}$ and $A_{C}$ represent the area of the surface, the hand (palm) and the parts of the hand and the surface that make contact.

The transfer rate between hands and various surfaces directly determines the amount of influenza A that is transmitted via the fomite route. Table 4 lists some values for the transfer rate between hands and surfaces with various materials.

Virus can reach the mucous membranes if a student touches his or her mouth, nasopharynx and eyes with a contaminated hand. Studies have shown that the mean rate of all finger contacts with the lips, nostrils and eyes ranges from 0.7 h^−1^ to 15 h^−1^ [36,51]. In this study, we assumed that the frequency of mucous membrane touching is 5 h^−1^. Therefore, from Table 1, one of four hand-face contacts are hand-mucous membrane contacts. The virus transfer rate from the fingertip to the mucous membranes is set to 35% [52]. The dose-response parameter based on intranasal inoculation of humans $\alpha_{I}=5.7\times 10^{-5}{\;\mathrm{TCID}}_{50}^{-1}$.

Resuspension of microorganisms from the floor, clothing and furniture acts as a secondary source [53]. Resuspended dust comprises up to 60% of the total particulate matter in indoor air [54,55]. The resuspension rate depends on many factors, such as the room height, the relative humidity and the particle size. To simplify the resuspension, we assumed that resuspension rate in the office is 10^−4^ h^−1^ [56]. Only resuspension between surfaces and the air is considered, while that between skin and the air is ignored because skin is usually moist.

Influenza A virus loses viability on surfaces over time. Porous and non-porous surfaces are both considered because the death rate of the virus differs significantly between the two surfaces [31]. According to a previous study [48], the inactivation rate of influenza A virus on a porous surface such as pyjamas ($\mu_{p}$) is 1.6 × 10^−2^ min^−1^, that on a non-porous surface such as stainless steel ($\mu_{s}$) is 2.0 × 10^−3^ min^−1^ and that on the hand ($\mu_{h}$) is 1.2 min^−1^. Inactivation on mucous membranes is not considered here.

Based on the balance between virus generation and disappearance (flew out of the room, lost viability or inhaled by people), the virus quantity in the air and on the surfaces can be calculated (Equations (5) and (6)).

(5) $$\frac{dV_{Air}\left(t\right)}{dt}=V_{Ps}+R_{SA}-D_{AS}-\frac{V_{Air}}{V_{R}}\cdot V_{AC}-\left(1-e^{-V_{Da}}\right)\cdot V_{Air}-V_{I},$$

(6) $$\frac{dV_{Sf}\left(t\right)}{dt}=V_{Pl}+D_{AS}-R_{SA}-\left(1-e^{-V_{Ds}}\right)\cdot V_{sf}\left(t\right)+V_{HS}-V_{SH},$$

where $V_{Air}$ and $V_{Sf}$ are the quantity of virus (TCID_50_) in the air and on surfaces in the office; $V_{Ps}$ and $V_{Pl}$ are the virus generation velocity in small droplets and large droplets, respectively, generated by breathing, talking, coughing and sneezing by the infected student; $D_{AS}$ and $R_{SA}$ are the particle deposition velocity from the air to surfaces and the resuspension velocity from surfaces to the air, respectively; $V_{R}$ is the room’s total volume; $V_{AC}$ is the air change rate; $V_{Da}$ and $V_{Ds}$ are the inactivation rate of virus in the air and on surfaces, respectively; $V_{HS}$ and $V_{SH}$ are the quantity of virus (TCID_50_) transferred from the hands to surfaces and from surfaces to the hands at each step, respectively; and $V_{I}$ is the inhalation velocity of each student.

### 2.4. Close Contact

Close contact is usually defined as being within 3 ft (~1 m) of the infector [36]. When two or more students talk, small and large droplets will be sprayed from the infected student’s mouth. Large droplets are likely deposited on the mucous membranes of susceptible students, some small droplets are directly inhaled by the students who are talking with the infected student and some of the remaining small droplets are inhaled by others via the long-range airborne route. In this study, a close contact was counted when a face to face contact occurs between any two students who are within 3 ft (~1m). The quantity of virus (TCID_50_) changes via inhalation and deposition on mucous membranes according to factors such as relative height, distance and direction in which the two students were facing and the room’s airflow. Here we assumed that 50% of small droplets [18,19] are inhaled by the student who is talking with the infected student and 30% of large droplets are deposited on that student’s face (10% of droplets on the face are deposited directly on the mucous membranes). Other small droplets diffuse into the air, and large droplets are deposited on surfaces nearby.

**Figure 2:**
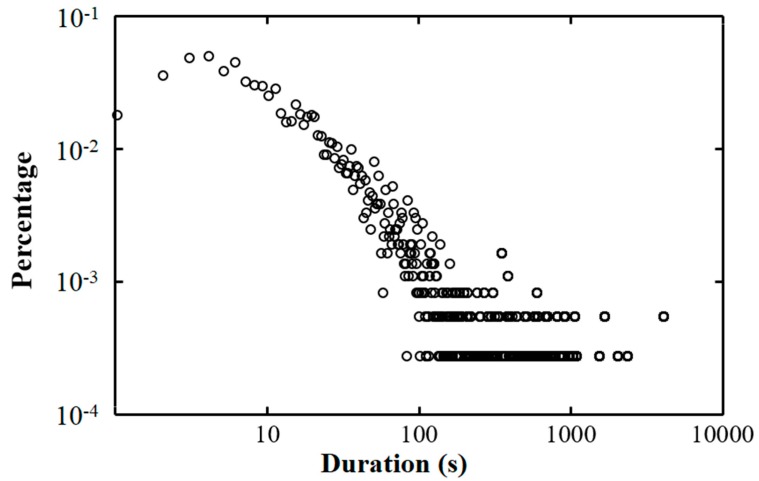
Association between the duration of close contact and the percentage of contacts.

We observed 3526 close contacts between students in an office over the course of 5 days. Figure 2 shows the association between the duration of close contact and percentage of contacts. The frequency of close contact per person is 9.64 h^−1^ per student, including active and passive contacts. Each student spent an average of 9.86% of their time in close contact. The average duration and the mean duration of close contact were 53.8 s and 17 s, respectively.

## 3. Results

### 3.1. Spatio-Temporal Virus Distribution

**Figure 3:**
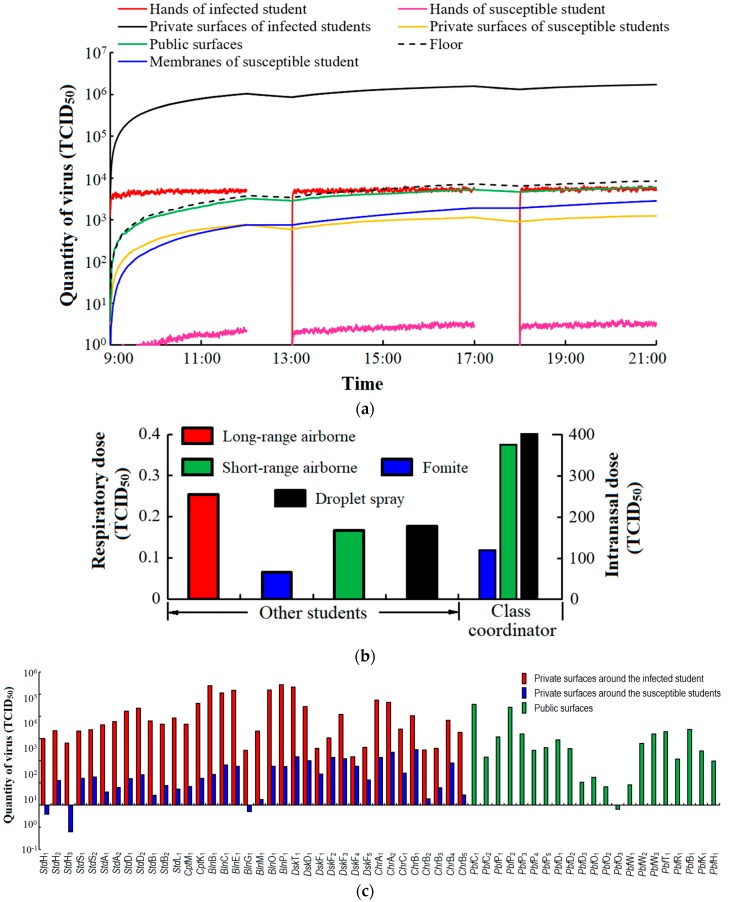
Quantity of virus (TCID_50_) (**a**) on different types of surface ^1^; (**b**) absorbed by the class coordinator and other students via various transmission routes (close contact includes both short-range airborne and droplet spray); (**c**) on different types of sub-surfaces after a whole day (TCID_50_ per surface).^1^ Quantity of virus (TCID_50_) on private surfaces, hands and mucous membranes of susceptible students are the average value of each student rather than a summation of all susceptible students.

Students move, make contact with other students, and touch surfaces in the office. Influenza A virus will be transferred between the hands and surfaces over time. From Figure 3a, the virus on the hands of the infected student increases rapidly and reaches a balance because limited numbers of his or her private surfaces share the virus. The hands of other students (susceptible) will be gradually contaminated, and virus on the hands of susceptible students almost reach a balance after 3 h (12 a.m.). The private surfaces of infected students are highly contaminated, and the quantity of virus (TCID_50_) on the private surfaces of the infected student is almost three orders of magnitude of that on the private surfaces of susceptible students. Public surfaces are dirtier than the private surfaces of susceptible students, and they also play important roles in the spread of infection like hubs in the surface touch network. The cumulative quantity of virus (TCID_50_) on the mucous membranes of susceptible students expresses each student’s intranasal dose and gradually increases over time.

The quantity of virus (TCID_50_) on the floor is relatively low because we assumed that most large droplets generated by talking, coughing and sneezing are deposited on the top surfaces of the desk, if the infected student is sitting in his or her own seat. In a 1-day simulation, we found that the respiratory dose per day of each susceptible student from the long-range and short-range airborne routes are 0.25 and 0.17 TCID_50_, respectively, and the intranasal dose per day from the fomite and droplet spray routes are 63.81 and 185.24 TCID_50_ (Figure 3b). All results are average values from 1000 simulations. Based upon the dose-response parameters from two routes, the total infection risk for each susceptible student during 1 day in the office is 8.75%, of which 54.31%, 4.23% and 44.46% are contributed by the long-range airborne, fomite and close contact routes. The class coordinator usually has more frequent interaction with other students, and we assumed that the probability of the class coordinator touching others’ desks and chairs and talking with others is twice that of the other students. The infection risk of the monitor is 13.79% (Figure 3b). The virus distribution on the surfaces of the desks and chairs of the class coordinator and the other students is shown in Figure S1 (Supplementary Materials). The class coordinator has a higher infection risk than the other students. There are 57 types of sub-surfaces, and the final quantity of virus (TCID_50_) on each type of sub-surface (TCID_50_ per surface) is shown in Figure 3c. The quantity of virus (TCID_50_) is much higher on the private surfaces around the infected student (approaching 800 times) than around susceptible students. Keyboards, headphones, desktops, mice and mobile phones are the five most-contaminated private surfaces around the infected student. The top of the seat back, the right chair arm, the desktop, the left chair arm and the top of the left desk’s fence are the five most-contaminated private surfaces around the susceptible students. Air conditioning (AC) controllers, printer touch screens, cabinet handles, tissue dispensers and the printer drawer are the dirtiest of all public surfaces. Surfaces with small areas, such as headphones and the buttons on the AC controller usually have a high virus concentration.

### 3.2. Infection Spread via the Fomite Route

**Figure 4:**
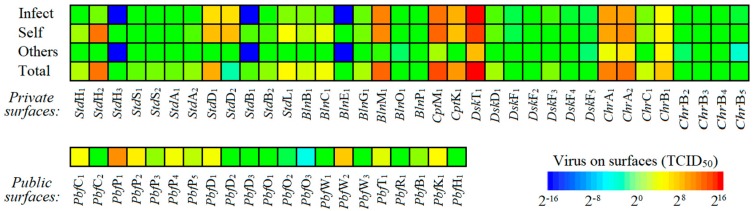
Quantity of virus (TCID_50_) transmitted from various types of sub-surfaces from the private surfaces of the infected student, the student’s own private surfaces, the private surfaces of other susceptible students and public surfaces to the hands (TCID_50_).

The spread of infection can be controlled only after the route of virus transmission is known. Figure 4 shows that 95.1% of virus is transmitted via private surfaces, while only 4.9% is transmitted via public surfaces. In all private surfaces, 59.8% of virus is transmitted via the private surfaces around the infected student, 38.1% of virus is transmitted via the private surfaces around the self and only 2.1% of virus is transmitted via the private surfaces of other susceptible students. The percentage of intranasal doses of susceptible students from the fomites of six primary surfaces—students, their belongings, computers, desks, chairs and public facilities—are 8.0%, 6.8%, 13.2%, 57.8%, 9.3% and 4.9%, respectively. Most virus absorbed by susceptible students comes from desktops, mice, mobile phones, faces, chair arms, keyboards, hands, printer touch screens and the button of the water dispenser. In addition, most virus absorbed by students comes from the private surfaces of infected students and the student himself or herself. The face is dirty, because some large droplets are deposited upon it when two students speak. Few students touch the faces of other students; therefore, the main sources of virus on the face are from the student himself or herself. During disinfection, the red and orange surfaces labelled in Figure 4 should receive more attention because of the high rate of virus transmission through them.

**Figure 5:**
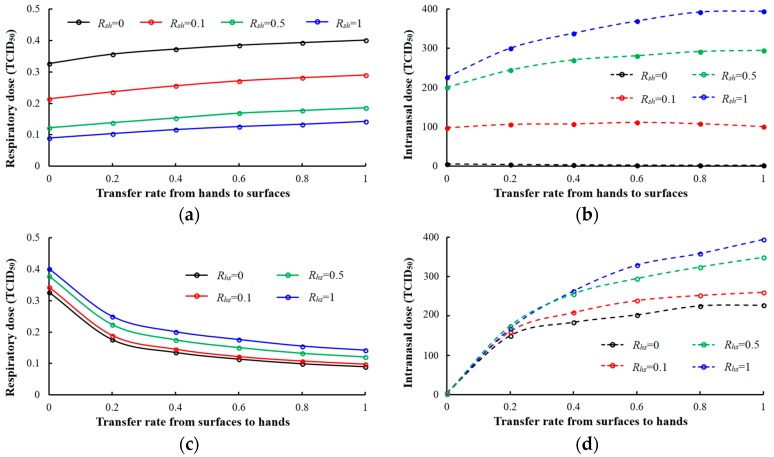
Different transfer rate between surfaces and hands ($R_{hs}$ & $R_{sh}$). (**a**) Respiratory dose changes with $R_{sh}$; (**b**) intranasal dose changes with $R_{sh}$; (**c**) respiratory dose changes with $R_{hs}$; (**d**) intranasal dose changes with $R_{hs}$.

Many factors influence effectiveness of virus transmission via fomites such as virus inactivation on surfaces and the virus transfer rate between hands and surfaces. With decreasing $R_{sh}$ and increasing $R_{hs}$, the respiratory dose per student per day gradually increases (Figure 5a). When $R_{sh}>0.1$, the intranasal dose increases as $R_{hs}$ increases, while when $R_{sh}<0.1$, the intranasal dose is negative in proportion with $R_{hs}$ (Figure 5b). When $R_{sh}$ is high, the quantity of virus (TCID_50_) on surfaces increases as $R_{hs}$ increases because more viruses are transferred from the hands of the infected student. When susceptible students touch the surfaces, more virus will be transferred from the surfaces to their hands. The intranasal dose of the susceptible students increases. When $R_{sh}$ is very low, it is difficult for the virus to transfer from surfaces to the hands. Although the virus from the hands of the infected student to surfaces increases as $R_{hs}$ increases, the transmission of virus from surfaces to the hands of susceptible students is limited because of small $R_{sh}$. As $R_{hs}$ increases, more virus is transmitted from the hands of susceptible students to surfaces than from the surfaces to their hands, and the intranasal dose of the susceptible students decreases. In reality, adjustment of $R_{sh}$ and $R_{hs}$ to a specific value can efficiently limit virus transmission via fomites, thus reducing the infection risk, especially in infectious diseases that are transmitted mainly via fomites such as norovirus. From Figure 5c,d, the respiratory dose gradually decreases as $R_{sh}$ increases and as $R_{hs}$ decreases. In contrast, the intranasal dose gradually increases, thus the total quantity of virus (TCID_50_) on surfaces decreases. The amount of virus aerosol in the air as a result of resuspension decreases. Therefore, in most cases, the respiratory dose decreases as intranasal dose increases.

### 3.3. Strategies for Influenza A Prevention

#### 3.3.1. Mask Wearing

**Figure 6:**
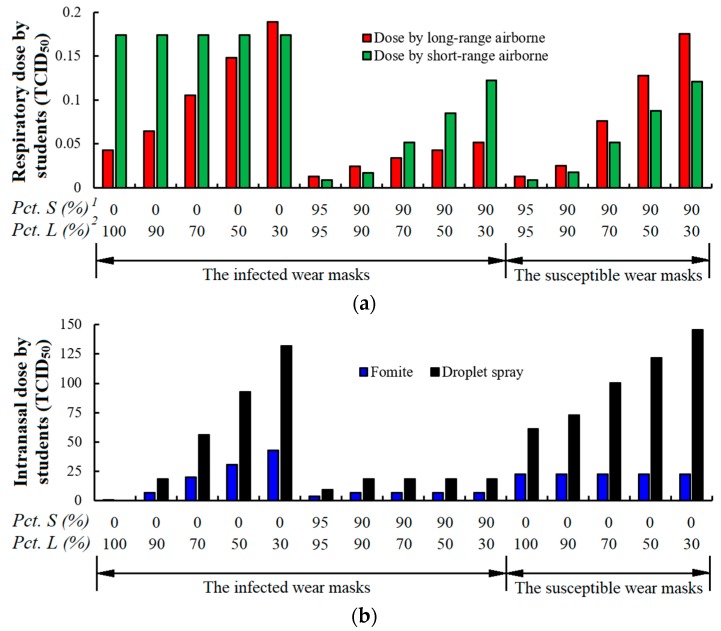
Dose of influenza A virus by susceptible students under different mask wearing strategies and efficiencies. (**a**) Long-range and short-range airborne routes; (**b**) fomite and droplet spray routes.^1^ Pct.S (%): Percentage of small particles blocked by masks.^2^ Pct.L (%): Percentage of large droplets blocked by masks.

Masks have various filtrating resolutions and efficacies. Surgical masks can only prevent large droplets, and N95 masks can prevent both large and small droplets. We hypothesised that 95% of both large and small droplets can be blocked by a tightly worn N95 mask. The particle block efficiency reduces if the mask is not tightly worn. Figure 6a,b show the respiratory and intranasal dose absorbed by susceptible students with different large and small droplets blocking efficiency and mask wearing strategies (the infected or the susceptible students wearing masks). When the blocking efficiency for large droplets ($E_{BL}$) is reduced from 100% to 30%, the respiratory dose of susceptible students from the long-range airborne route increases from 0.04 to 0.19 TCID_50_. The intranasal dose via fomites and droplet spray increase from 0.01 to 42.53 TCID_50_ and from 0 to 131.79 TCID_50_, respectively (Figure 6b). When $E_{BL}=100\%$, the intranasal dose comes only from the deposition of small aerosol in the air. Therefore, comparing no mask with wearing mask with 100% $E_{BL}$, each susceptible student’s average infection risk is reduced from 8.75% to 3.82%. When an N95 mask is worn, small droplets can also be filtered. By increasing the mask’s blocking efficiency for small droplets ($E_{BS}$), the respiratory dose via both long-range and short airborne routes decreases (Figure 6a). However, the intranasal dose via the fomite and droplet spray routes remain nearly the same if $E_{BS}$ increases (Figure 6b). When 95% of both small and large droplets are blocked ($E_{BL}=E_{BS}=95\%$), the infected student’s total risk of infection will be reduced from 8.75% to 0.45%.

If only susceptible students wear the mask rather than the infected student, the infection risk changes. The respiratory dose via the long-range and short-range airborne routes decreases with increasing $E_{BS}$. When 95% of both small and large droplets are blocked, the respiratory dose from the airborne route is reduced to 0.02 TCID_50_ (Figure 6a). A mask can block the virus from the hands to the nose and lips because the mask isolates them. When all susceptible students wear masks with high $E_{BL}$, intranasal dose through fomites can hardly be further reduced. This case differs when only the infected student wears the high-$E_{BL}$ mask. The intranasal dose caused by droplet spray decreases as $E_{BL}$ increases (Figure 6b). The total infection risk will be reduced to 0.87% if all susceptible students tightly wear N95 masks.

#### 3.3.2. Ventilation

**Figure 7:**
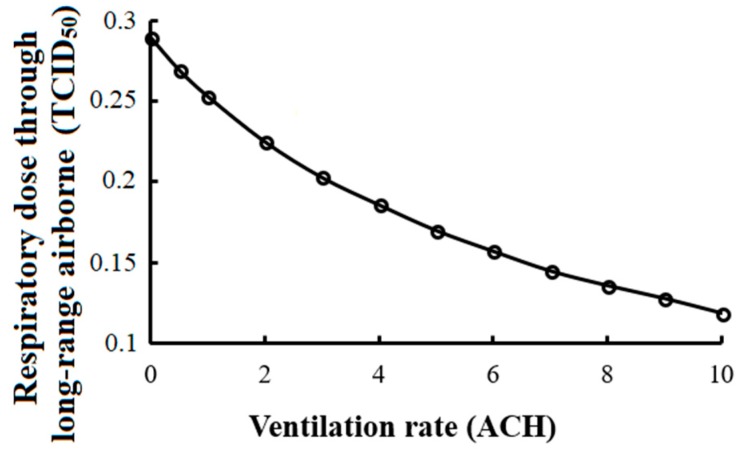
Respiratory dose via the long-range airborne route at various ventilation rates.

It is well known that ventilation can efficiently reduce the risk of infection via the long-range airborne route. It is determined by natural and mechanical ventilation, which are influenced by many factors such as open doors, open windows and AC systems. When the ventilation rate changes from 1 ACH to 4 ACH, the respiratory dose via the long-range airborne route will be reduced from 0.26 to 0.19 TCID_50_, and the general infection risk will be reduced by 1.2% (Figure 7). If the ventilation rate increases to 10 ACH, the infection risk via the long-range airborne route will be less than half under only 1 ACH. With no ventilation, all virus in the air moves out only through inactivation and deposition, and the virus concentration in the air is much higher.

#### 3.3.3. Hand Washing

**Figure 8:**
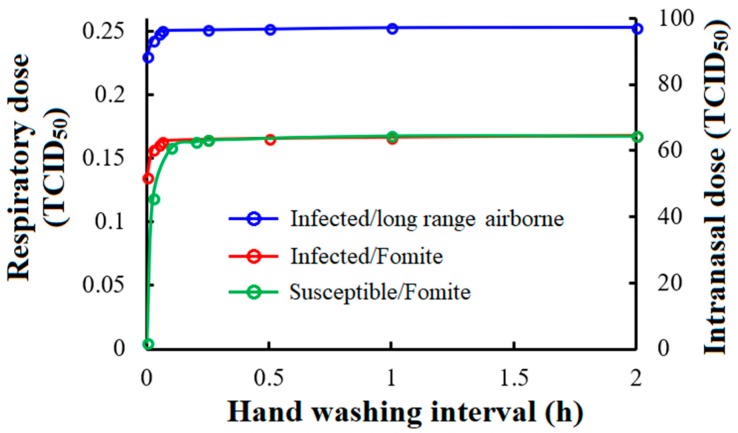
Infection risk reduction by hand washing (Blue and red lines show the respiratory and intranasal doses of the susceptible students when only the infected student washes his or her hands; the green line shows the intranasal dose from susceptible students when only they wash hands).

Figure 8 shows how hand washing by the infected student and susceptible students influences the respiratory and intranasal dose. In the simulation, we hypothesised that hands become completely clean (no virus) after hand washing. The infected student’s hands usually possess a large quantity of virus (TCID_50_), which will then be transmitted to surfaces and resuspended in the air. As shown in blue and red lines in Figure 8, when the infected student’s hand washing frequency is less than 6 times per hour, the decrease in the respiratory and intranasal doses for susceptible students is obvious. If susceptible students wash their hands, rather than the infected student, the infection risk reduction via fomites is slightly higher. If the hand washing frequency is less than 6 times per hour, the intranasal dose reduction via fomites will be obviously limited. Moreover, hand washing by susceptible students hardly reduces the respiratory dose. In general, focusing on the influenza A virus, if the frequency of hand washing is more than twice per hour, very little further improvement can be made in the efficacy for infection risk reduction.

#### 3.3.4. Surface Cleaning

**Figure 9:**
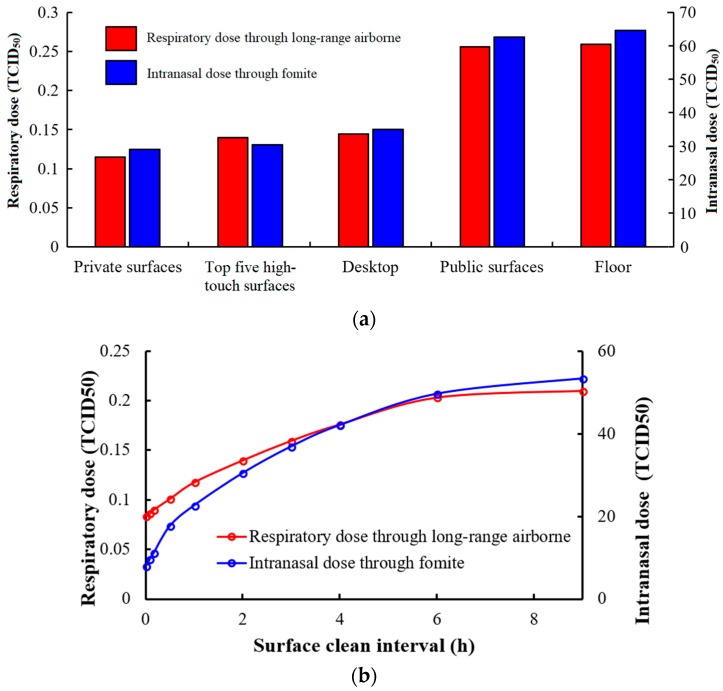
Surface cleaning. (**a**) Various types of surfaces with a cleaning frequency of 0.5 h−1; (**b**) different clean interval for top five high-touch surfaces ^1^. ^1^ Top five high-touch surfaces: desktop, mouse, mobile phone, keyboard and chair arms.

In the simulation, we hypothesised that surfaces become completely clean (no virus) after surface cleaning. Figure 9a shows how respiratory and intranasal doses change with various strategies of surface cleaning with frequency of 0.5 h^−1^. No student touches the floor, and a clean floor can only reduce the resuspension of aerosol. Regular desktop cleaning can reduce the respiratory and intranasal dose from 0.26 to 0.14 TCID_50_ and from 65.87 to 35.10 TCID50, respectively (the general infection risk ranges from 8.75% to 6.71%). The cleaning of more surfaces, such as the top five high-touch surfaces and all private surfaces, can slightly reduce the infection risk from 6.71% to 6.61%, and to 6.17%, respectively.

Figure 9b shows that the respiratory and intranasal dose grows logarithmically as the interval at which high-touch surfaces are cleaned increases. If only a regular high-touch surface cleaning is conducted at 3 p.m. each day, the respiratory dose via long-range airborne and the intranasal dose via fomites are 0.16 and 36.97 TCID_50_ (infection risk = 6.97%), respectively. However, if the frequency of surface cleaning is increased to once per hour (surface clean interval = 1 h), the infection risk is reduced to 6.19%. With a continuous decrease in the clean frequency when it is lower than once per hour, the infection risk will be reduced quickly, but the workload is huge.

## 4. Discussion

In this paper, we studied how influenza A transmits in a graduate student office in one day from 9 a.m. to 9 p.m. through long-range airborne, fomite and close contact (short-range airborne and droplets spray) routes based on real recorded data of contacts between students and surfaces touch. Most parameters set in the simulation are from real recorded data and existing studies.

We found that each susceptible student’s average infection risk during a day in the office is 8.75%, of which 54.31%, 4.23%, 33.24% and 11.22% are contributed by the long-range airborne, fomite, short-range airborne and droplet spray routes. However, in an air cabin, focusing on influenza A H1N1, the contributions via the airborne, close contact and fomite routes are 34.30%, 64.98% and 0.72% [13]. In the air cabin, close contact is more frequent because of the high density of passengers. Fomites contribute less due to the low probability of direct touch between the infected and susceptible passengers. The long-range airborne route is less frequent because a high ventilation rate (25 ACH) is set in the air cabin even though the population density is high. Focusing on virus on surfaces, the private surfaces around the infected student had the most virus, because most contaminated droplets are deposited on surfaces around the infected person, and nearby surfaces are also easily touched [18]. The quantity of virus (TCID_50_) on all surfaces stops its rapid increase after 3 h. Virus on the hands of susceptible students always remains at a lower level because the inactivation rate of influenza A on the hands is high. The quantity of virus (TCID_50_) on public surfaces is higher than on private surfaces around the susceptible students. Moreover, some active students such as the class coordinator have more contacts and a higher probability of touching other’s private surfaces. We found that the class coordinator’s infection risk is almost 1.6 times that of other students.

Approximately 4.2% of the influenza A infection risk comes from fomites. However, for other infectious diseases such as norovirus, which makes more than 85% of the contribution to infection risk [13], and fomite plays an important role. In the office, 95.1% of viruses were transmitted via private surfaces and only 4.9% via public surfaces. The desk is the dirtiest of all private surfaces. In this study, we hypothesised that most contaminated droplets generated by talking, coughing and sneezing are deposited on desks. Desktops, mice, mobile phones and keyboards transmit virus easily. This result accords with previous studies on microbiome [57,58]. Public surfaces are the hubs of the surface touch network [16]. AC controller buttons, printer touch screens and public cabinet handles are highly contaminated. The button of the water dispenser is frequently touched. However, the infected students usually touched the chair arms, cups and seatbacks of other students before getting a cup of water. The virus on hands is diluted, and the total quantity of virus (TCID_50_) on the water dispenser is limited. These high-risk surfaces should be given more attention in infectious disease transmission, especially on some diseases that are highly dependent on fomite, such as norovirus. If we can design an anti-virus material to build all high-touch surfaces, we could efficiently control virus transmission via fomites.

From a virus transfer rate perspective, if we can create a surface that can block virus transmission from surfaces to hands ($R_{sh}=0$), the virus from fomites could be efficiently controlled. Indeed, the transfer rate between surfaces and hands cannot reach 0 or 1. A material with low $R_{sh}$ can limit virus transmission from surfaces to hands, such as porous materials [44]. Surfaces made with material with a high $R_{hs}$ can absorb more virus from hands of both the infected student and susceptible students. Therefore, the means by which to adjust the virus transfer rate between surfaces and hands by using materials with different $R_{sh}$ and $R_{hs}$ is very helpful to prevent virus transmission via fomites. We found that when $R_{sh}>0.1$, the intranasal dose increases as $R_{hs}$ increases, while when $R_{sh}<0.1$, it decreases with $R_{hs}$ in an office setting.

Many strategies, such as mask wearing [59], ventilation [4], hand washing [11] and surface cleaning [60], can limit the spread of influenza A in a confined room. Wearing a mask can control the spread of disease via the long-range airborne, fomite and close contact routes. A high ventilation rate helps to dilute the virus concentration in the air, and the respiratory dose from the long-range airborne route will be obviously reduced. Hand washing can reduce the infection risk directly via fomites and indirectly via the long-range airborne route because of the lower rate of resuspension from surfaces to the air. The same effects are brought by surface cleaning. Mask wearing is much more efficient than hand washing because influenza A is transmitted mainly via the airborne and close contact routes. Mask wearing by the infected student has greater efficiency at reducing the infection risk than simply having the susceptible students wear masks (infection risk of 0.87% vs. 0.45% when an N95 mask is tightly sealed). According to the test in hospitals, tightly sealing a mask to the face can block the entry of 94.5% of total virus and 94.8% of infectious virus [59]. We found that 94.9% of the infection risk can be reduced if an N95 mask is tightly sealed on the infected student, which is very useful in influenza A transmission control. The airborne route is the main route of influenza A virus spread. In a norovirus outbreak, the efficiency of mask-wearing should be reassessed.

Hands play a very important role in the surface touch network, because hands can be contaminated by covering the mouth and nose when coughing and sneezing or by touching contaminated surfaces, and thus can also contaminate surfaces. Some previous studies have shown that hand washing can cut the risk of respiratory infection by 16% [61]. We found that only hand washing is limited to reduce infection risk. When the hand washing frequency is greater than six times per hour, the infection risk can be obviously reduced. However, in the hospital, when all doctors and nurses wear masks, hand washing is more efficient because hands are one of the main ways to spread virus from hospital workers to susceptible people. We also found that if the susceptible students wash their hands (rather than the infected student), the efficiency of infection risk reduction is slightly higher.

Students spent more than 90% of their time in the office touching surfaces. The intranasal dose due to contact between hands and mucous membranes depends on the quantity of viable pathogens on the office’s surfaces, the frequency of contact between hands and contaminated surfaces, contact between hands and mucous membranes and the efficiency of pathogen transfer to and from hands-on contact [18]. Regular surface cleaning can reduce the infection risk. Public surfaces are frequently touched, so viruses on public surfaces are diluted by many touches by susceptible students. Public surface cleaning every 2 h is not very efficient because there are few public surfaces in the office. Desktops are among the most contaminated surfaces in the office because the students spent most of their time at their desks. Contaminated droplets are deposited on the desktop when an infected student talks, coughs or sneezes. Students often touch the desktops of other students, because of the high frequency of discussions between students in the office. Cleaning of all private surfaces can reach a better condition (infection risk reduced by 2.56%), but the workload is too large. Therefore, regular cleaning of desktops or high-touch surfaces is suggested if the virus is very severe. In addition, cleaning of high-touch surfaces is much more efficient than hand-washing because high-touch surfaces gathered more virus and because the inactivation rate on surfaces is much lower than on the hands. The results of influenza A simulation based on real data of human behaviour in a confined space is very helpful to help understand the real characteristics of influenza A transmission and to make effective plans to prevent and control diseases.

This work perhaps lacks a direct and strong connection to Health-EDRM (health-related emergency disaster risk management). However, the model that we built and suggestions that we put forward are useful in for controlling infectious diseases transmission, and major outbreaks if infectious diseases can be itself a health-related disaster. Moreover, natural and complex disasters such as floods, tsunamis, and earthquakes can dramatically increase the risk of infectious diseases outbreak including malaria, measles, viral hepatitis, etc. [62,63]. After a serious disaster, many people who have been affected by the disaster will be gathered in an emergency shelter. Risk of infectious disease transmission through different routes can be calculated based on our model, and suggestions we obtained also can guide disaster managers in making efficient emergency plans for infectious disease control and prevention after disasters.

This study has various limitations. All students are assumed to stay in the office at all times except at lunch and dinner time, virus generation is overestimated, and a higher infection risk of susceptible students is calculated. The virus transfer rate between surfaces and hands is influenced by many factors, such as force, area and touch duration. We assumed that each touch between a specific surface and a hand has the same transfer rate. Most particles are deposited on the desktop when the infected student talks, coughs or sneezes, and we did not consider that some private things such as mice, keyboards and cups share the virus on the desk. Therefore, the quantity of virus (TCID_50_) on the desk is overestimated. Moreover, in our simulation, some parameters such as transfer rate between hand and various surfaces are not based on influenza virus due to data unavailability. Heterogeneity exists in the study, and it may result in some errors. The differences in human behaviour by gender are ignored, and relative positions, heights and angles between two students during close contact are not considered. A future study should collect some samples in the office if any students are infected with influenza A. We can then compare the real virus distribution data with our simulation data to verify the accuracy of our model.

## 5. Conclusions

Influenza A transmission in a graduate student office is simulated via long-range airborne, fomite, and close contact routes based on realistic data of human behaviours. The long-range airborne, fomite and close contact routes contribute to 54.3%, 4.2% and 44.5% of influenza A infections, respectively. For the fomite route, 59.8%, 38.1% and 2.1% of viruses are transmitted to the hands of students from private surfaces around the infected students, the students themselves and other susceptible students, respectively. The private surfaces of infected students are highly contaminated. The quantity of virus (TCID_50_) is much higher on the private surfaces around the infected student (approaching 800 times) than around susceptible students. Keyboards, headphones, desktops, mice and mobile phones are the five most-contaminated private surfaces around the infected student. Public surfaces are dirtier than the private surfaces of susceptible students. The intranasal dose via fomites of the students’ bodies, belongings, computers, desks, chairs and public facilities are 8.0%, 6.8%, 13.2%, 57.8%, 9.3% and 4.9%, respectively. The intranasal dose does not monotonously increase or decrease with the virus transfer rate between hands and surfaces, and a specific value setting can optimally limit influenza A virus transmission via fomites Mask wearing is much more useful than hand washing for control of influenza A in the tested office setting, and the total risk can be reduced from 8.75% to 0.45% if an N95 mask is tightly sealed by infected students. Regular cleaning of high-touch surfaces, which can reduce the infection risk by 2.14%, is recommended and is much more efficient than hand-washing.

## Figures and Tables

**Table 1:** Modes of person-to-person transmission of respiratory viruses [20].

Transmission Route	Definition
Aerosol transmission	Virus is transmitted through the air by aerosols within the inspirable size range or smaller; aerosol particles are small enough to be inhaled into the nasopharynx and distally into the trachea and lung.
Direct transmission	Virus is transferred by contact from an infected person to another person without a contaminated intermediate object (fomite).
Indirect transmission	Virus is transferred by contact with a contaminated intermediate object (fomite).
Droplet spray transmission	Virus is transmitted through the air by droplet sprays (such as those produced by coughing or sneezing); a key feature is deposition of droplets by impaction on exposed mucous membranes.

**Table 2:** Parameters related to long-range airborne.

Parameter	Symbol	Value	Source
Deposition rate	*R_D_*	0.1 min^−1^	Assumed
Volume of small particles by breathing	$V_{vb}^{\prime }$	$1.02\times 10^{-10}\;\mathrm{mL}$	[23,24,25,26,27,28,29]
Volume of small particles by talking	$V_{vt}^{\prime }$	$1.47\times 10^{-7}\;\mathrm{mL}$	[23,24,25,26,27,28,29]
Volume of small particles by coughing	$V_{vc}^{\prime }$	$1.65\times 10^{-7}\;\mathrm{mL}$	[23,24,25,26,27,28,29]
Volume of small particles by sneezing	$V_{vs}^{\prime }$	$1.27\times 10^{-6}\;\mathrm{mL}$	[23,24,25,26,27,28,29]
Volume of large droplets by breathing	$V_{vb}^{\prime \prime }$	0 mL	[23,24,25,26,27,28,29]
Volume of large droplets by talking	$V_{vt}^{\prime \prime }$	$5.15\times 10^{-3}\;\mathrm{mL}$	[23,24,25,26,27,28,29]
Volume of large droplets by coughing	$V_{vc}^{\prime \prime }$	$6.15\times 10^{-3}\;\mathrm{mL}$	[23,24,25,26,27,28,29]
Volume of large droplets by sneezing	$V_{vs}^{\prime \prime }$	$4.75\times 10^{-2}\;\mathrm{mL}$	[23,24,25,26,27,28,29]
Frequency of breath	*F_b_*	15 min^−1^	[30]
Frequency of coughing (infected person)	*F_c_*	22 h^−1^	[31]
Frequency of sneezing (infected person)	*F_s_*	5 h^−1^	[32]
Duration per cough	*D_c_*	1 s	Assumed
Duration per sneeze	*D_s_*	3 s	Assumed
Percentage of time on talking with others	*P_t_*	10%	Monitored
Viral shedding rate	*R_s_*	$1.5\times 10^{7}\;\mathrm{TCID}50/\mathrm{day}$	[19,24,25,31,33]
Virus concentration in exhaled particles	*C_v_*	$1\times 10^{6.39}\;\mathrm{TCID}50/\mathrm{mL}$	Calculated based on [19,23,24,25,26,27,28,29,31,33,34]
Ventilation rate in the office	*V_AC_*	1 ACH	[37]
Respiratory rate of a person	*R_R_*	0.38 m^3^/h	[38]
Inactivation rate of influenza A ^1^	$\mu_{a}$	13.9 day^−1^	[40]
Dose-response parameter	$\alpha_{R}$	$0.18\;\mathrm{TCID}50^{-1}$	[36]

**^1^** The inactivation rate of influenza A in aerosols in the air in the condition of 50% R.H and 20 °C–24 °C.

**Table 3:** Detailed information of all sub-surfaces.

Sub-Surf ^1^	Surf Type ^2^	F ^3^ (h^−1^)	Area ^4^ (cm^2^)	Hori-Coef ^5^	Sub-Surf ^1^	Surf Type ^2^	F ^3^ (h^−1^)	Area ^4^ (cm^2^)	Hori-Coef
*Std*H_1_	S	6.32	469	0.5	*Std*H_2_	S	19.41	470	0
*Std*H_3_	S	1.01	470	0	*Std*S_1_	P	0.28	504	0.5
*Std*S_2_	P	0.34	504	0.5	*Std*A_1_	P	1.17	1293	0.5
*Std*A_2_	P	1.35	1293	0.5	*Std*D_1_	S	3.76	183	0
*Std*D_2_	S	3.80	183	0	*Std*B_1_	P	2.86	2401	0
*Std*B_2_	P	0.99	940	0	*Std*L_1_	P	12.64	6592	0
*Bln*B_1_	P	3.05	2600	0.5	*Bln*C_1_	N	2.88	500	0
*Bln*E_1_	N	1.94	10	0.5	*Bln*G_1_	N	2.71	10	0.5
*Bln*M_1_	N	15.12	200	0.5	*Bln*O_1_	P	0.65	7500	0
*Bln*P_1_	P	0.39	2400	0.5	*Cpt*M_1_	N	23.61	120	0.5
*Cpt*K_1_	N	28.98	700	1	*Dsk*T_1_	N	20.19	6000	1
*Dsk*D_1_	St	1.04	90	0.25	*Dsk*F_1_	N	0.08	300	1
*Dsk*F_2_	N	0.14	180	1	*Dsk*F_3_	N	0.18	180	1
*Dsk*F_4_	N	0.06	400	0	*Dsk*F_5_	N	0.06	400	0
*Chr*A_1_	N	3.17	200	1	*Chr*A_2_	N	3.00	200	1
*Chr*C_1_	P	0.73	1200	1	*Chr*B_1_	P	1.56	100	1
*Chr*B_2_	P	0.09	1200	0	*Chr*B_3_	P	0.23	1200	0
*Chr*B_4_	P	0.09	80	0	*Chr*B_5_	P	0.05	80	0
*Pbf*C_1_	N	0.03	5	0	*Pbf*C_2_	N	0.01	60	0
*Pbf*P_1_	N	<0.01	200	1	*Pbf*P_2_	N	0.28	35	0
*Pbf*P_3_	N	0.08	100	0	*Pbf*P_4_	N	0.51	3200	0
*Pbf*P_5_	N	0.09	660	0	*Pbf*D_1_	St	0.05	100	0.25
*Pbf*D_2_	N	0.04	300	0	*Pbf*D_3_	N	0.01	5000	0
*Pbf*O_1_	St	<0.01	100	0.25	*Pbf*O_2_	St	0.01	1600	0
*Pbf*O_3_	N	<0.01	6000	0	*Pbf*W_1_	N	0.01	12000	0
*Pbf*W_2_	N	0.05	2750	0.2	*Pbf*W_3_	N	0.31	12	1
*Pbf*T_1_	N	0.18	200	0	*Pbf*R_1_	N	0.04	400	0
*Pbf*B_1_	N	0.14	10	0.5	*Pbf*K_1_	N	0.08	4400	1
*Pbf*H_1_	N	0.03	4260	0.4					

^1^ Sub-surf: sub-surfaces. The codes of 57 sub-surfaces can be found in Table S1. ^2^ Surf type: type of surfaces including porous (P), non-porous (N), stainless steel (St) and skin (S). No one wears shorts while in the office, so the hands, head, face and neck are regarded as skin, and other body parts are regarded as porous surfaces. ^3^ Frequency of each sub-surface to be touched (h^−1^). ^4^ Data for the human body’s skin area were obtained from [42,43]. The area of the hand does not include the back of the hand. Area for other surfaces was measured or estimated. Therefore, the area considered includes only the parts usually touched by hands; for example, the area of a mouse includes only the top and side surfaces because few people will directly touch its bottom surface. ^5^ Horizontal coefficient of area. It means that the portion of the area that is horizontal can gather particles caused by deposition. For example, a horizontal coefficient of 1 means that all surface areas are horizontal and are totally exposed to particle deposition (e.g., *Dsk*T1: desktop).

**Table 4:** **^1^** Virus transfer rate between hands and surfaces.

Donor	Recipient	Transfer Rate	Donor	Recipient	Transfer Rate
Porous	Hand	3% [44]	Hand	Porous	80% [45]
Non-porous	Hand	7% [46]	Hand	Non-porous	12% [47]
Stainless steel	Hand	7.9% [48]	Hand	Stainless steel	16.1% [49]
Hand	Hand	25.5% [50]			

^1^ All surfaces in the office are regarded as composed by materials shown in the Table. In addition, many factors influence the transfer rate, including temperature, humidity, touch duration and dry/wet hand, thus causing some errors here.

## Supplementary Materials

The following are available online at http://www.mdpi.com/1660-4601/15/8/1699/s1, Figure S1: Surfaces contamination around the class coordinator and other students, Table S1: Types of surfaces in student office.
