# Supplementary material for: Transmission of Influenza A in a Student Office Based on Realistic Person-to-Person Contact and Surface Touch Behaviour
Source: Int J Environ Res Public Health. 2018 Aug 9;15(8):1699. doi: 10.3390/ijerph15081699 (PMC6121424; doi:10.3390/ijerph15081699)
Supplement: Supplementary file 1 [file ijerph-15-01699-s001.pdf]

Supplementary Materials

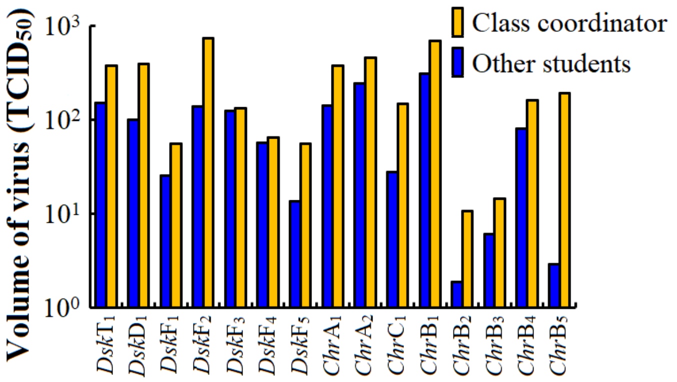

Figure S1. Surfaces contamination around the class coordinator and other students.

Table S1. Types of surfaces in student office.

Primary surface	Secondary surface	Code	Sub-surface	Code
			Head	*Std*H_1
	Head	*Std*H	Face	*Std*H_2
			Neck	*Std*H_3
	Shoulder	*Std*S	Left shoulder	*Std*S_1
			Right shoulder	*Std*S_2
**Students (*Std*)**	Arm	*Std*A	Left arm	*Std*A_1
			Right arm	*Std*A_2
	Hand	*Std*D	Left hand	*Std*D_1
			Right hand	*Std*D_2
	Body	*Std*B	Front	*Std*B_1
			Back	*Std*B_2
	Hip, leg, foot	*Std*L	Hip & leg	*Std*L_1
	Bag	*Bln*B	Bag	*Bln*B_1
	Cup	*Bln*C	Cup	*Bln*C_1
	Headphones	*Bln*E	Headphones	*Bln*E_1
**Belongings (*Bln*)**	Frame of glasses	*Bln*G	Frame of glasses	*Bln*G_1
	Mobile phone	*Bln*M	Mobile phone	*Bln*M_1
	Overcoat	*Bln*O	Overcoat	*Bln*O_1
	Pillow	*Bln*P	Pillow	*Bln*P_1
**Computer (*Cpt*)**	Mouse	*Cpt*M	Mouse	*Cpt*M_1
	Keyboard	*Cpt*K	Keyboard	*Cpt*K_1
	Desktop	*Dsk*T	Desktop	*Dsk*T_1
	Drawer	*Dsk*D	Drawer	*Dsk*D_1
			Top of front fence	*Dsk*F_1
**Desk (*Dsk*)**			Top of left fence	*Dsk*F_2
	Fence	*Dsk*F	Top of right fence	*Dsk*F_3
			Side of left fence	*Dsk*F_4
			Side of right fence	*Dsk*F_5

**Chair (*Chr*)**	Arm	*Chr*A	Left chair arm	*Chr*A_1
			Right chair arm	*Chr*A_2
	Cushion	*Chr*C	Cushion	*Chr*C_1
	Seat back	*Chr*B	Top of seat back	*Chr*B_1
			Back of seat back	*Chr*B_2
			Front of seat back	*Chr*B_3
			Left of seat back	*Chr*B_4
			Right of seat back	*Chr*B_5
**Public facility (*Pbf*)**	Controller	*Pbf*C	AC controller	*Pbf*C_1
			Light switch	*Pbf*C_2
	Printer	*Pbf*P	Cover	*Pbf*P_1
			Screen	*Pbf*P_2
			Drawer	*Pbf*P_3
			Body	*Pbf*P_4
			Scanning surface	*Pbf*P_5
	Door	*Pbf*D	Handle	*Pbf*D_1
			Side	*Pbf*D_2
			Main surface	*Pbf*D_3
	Window	*Pbf*O	Handle	*Pbf*O_1
			Frame	*Pbf*O_2
			Glass	*Pbf*O_3
	Water dispenser	*Pbf*W	Body	*Pbf*W_1
			Bucket	*Pbf*W_2
			Button	*Pbf*W_3
	Tissue dispenser	*Pbf*T	Tissue dispenser	*Pbf*T_1
	Trash can	*Pbf*R	Trash can	*Pbf*R_1
	Cabinet handle	*Pbf*B	Cabinet handle	*Pbf*B_1
	Desk beneath printer	*Pbf*K	Desk beneath printer	*Pbf*K_1
	Chair in front of printer	*Pbf*H	Chair in front of printer	*Pbf*H_1
